# Cognitive Training Mobile Apps for Older Adults With Cognitive Impairment: App Store Search and Quality Evaluation

**DOI:** 10.2196/69637

**Published:** 2025-07-04

**Authors:** Leyi Wu, Jiajuan Pan, Chuwen Dou, An Gu, An Huang, Hong Tao, Xiaoyan Wang, Chen Zhang, Lina Wang

**Affiliations:** 1 School of Medicine, Huzhou Key Laboratory of Precise Prevention and Control of Major Chronic Diseases, Huzhou University, Erhuan East Road 759, Longquan Street, Wuxing District, Huzhou, 313000, China, 86 13587278357; 2 Center for Whole-Person Research, AdventHealth Whole-Person Research, Orlando, FL, United States; 3 Department of General Medicine, Community Health Service Center of Renhuangshan, Huzhou, China

**Keywords:** cognitive training, older adults, mobile apps, mobile app rating scale, eHealth

## Abstract

**Background:**

As the population ages, cognitive impairment is becoming increasingly prevalent. Mobile apps offer a scalable platform for delivering cognitive training interventions. However, their variable quality and lack of rigorous evaluation underscore the need for further research to guide optimization and ensure their effective application in improving cognitive health outcomes.

**Objective:**

This study aimed to evaluate the characteristics and quality of cognitive training apps designed for older adults with cognitive impairment.

**Methods:**

A comprehensive search of the Google Play Store and Apple App Store was conducted using predefined terms and inclusion criteria, with the search completed on July 13, 2024. Eligible apps were assessed for quality by two independent reviewers using the Mobile App Rating Scale (MARS), with interrater reliability evaluated via quadratic weighted kappa (К). The Kruskal-Wallis test analyzed differences in MARS scores across subgroups for each dimension, and Spearman correlation was applied to examine the relationship between user star ratings and overall mean scores.

**Results:**

A total of 4822 potential apps were identified, of which 24 met eligibility criteria. Among these, 13 (54%) were available on both platforms, 5 (21%) were exclusive to the Google Play Store, and 6 (25%) to the Apple App Store. Notably, 5 (20.8%) apps offered user-tailored training modules and 8 (33%) involved medical professionals in development. Interrater agreement was high (k=0.88; 95% CI, 0.80‐0.95). Global quality scores based on the MARS dimensions ranged from 2.38 to 4.13, with a mean (SD) of 3.57 (0.43) across 24 apps, indicating generally acceptable quality. The functionality dimension received the highest score, while engagement scored the lowest. Brain HQ and Peak had scores above 4 and were rated as good, whereas Memory Trainer, Cognitive Skill Training, and Ginkgo Memory & Brain Training scored below 3 and were rated as insufficient. Spearman correlation showed no significant association between mean score and app rating.

**Conclusions:**

Current cognitive training apps for older adults with cognitive impairment demonstrate moderate quality with considerable variability. Improvements are needed in the engagement and information dimensions. Future development should prioritize enhancing user engagement, incorporating personalized features, and involving health care professionals and experts to align with evidence-based guidelines.

## Introduction

The global aging population presents a significant public health challenge due to the increasing prevalence of dementia. Aging is the primary risk factor for dementia, which affects an estimated 50 million individuals worldwide, with projections reaching 152 million by 2050 [1]. Age-related processes, including neurofibrillary tangles, amyloid-beta plaque deposition, and cerebrovascular changes, contribute to the pathogenesis of Alzheimer disease and other dementias [2]. Therefore, efforts to address cognitive decline in older adults are critical to mitigating the growing global burden of dementia.

**Figure 1.**
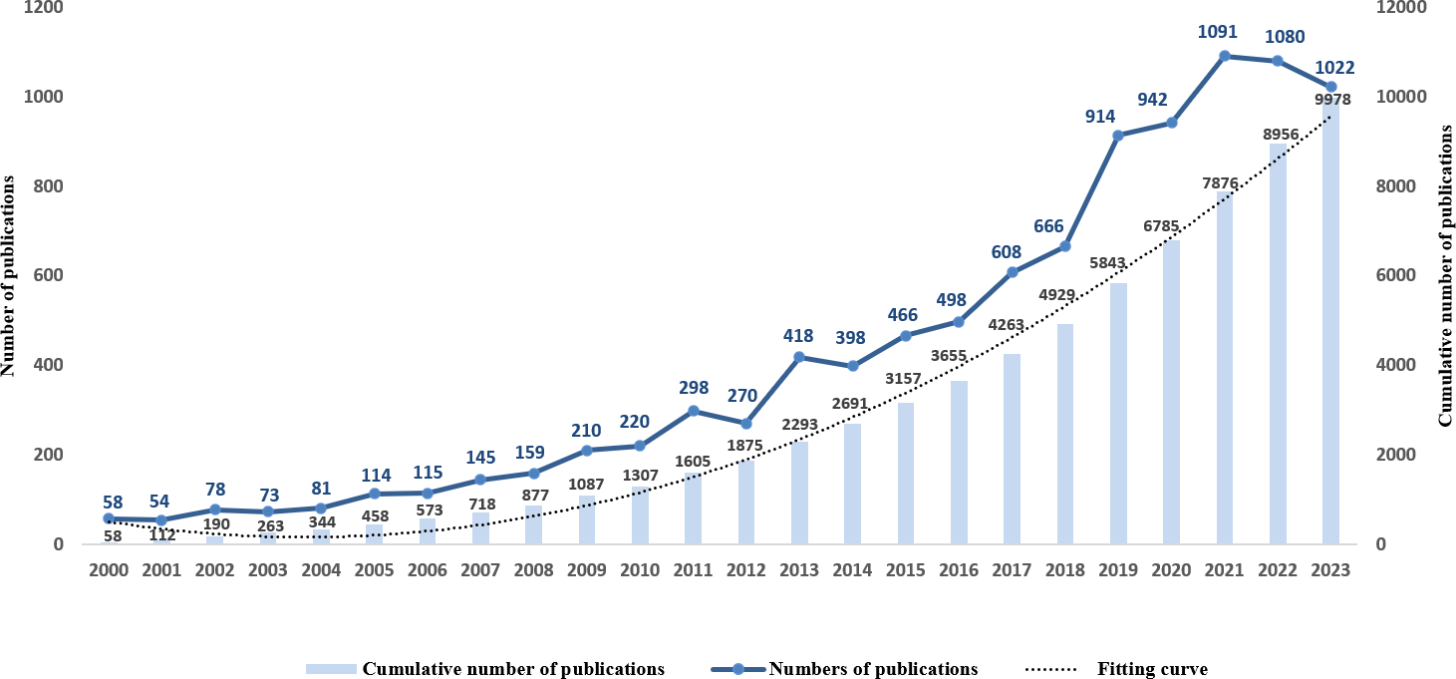
Trends in annual and cumulative number of publications on cognitive training (2000‐2023).

Cognitive interventions have emerged as a promising strategy for maintaining cognitive function in older adults, particularly those with cognitive impairment. The popularity and development of digital cognitive training technology as one of the key applications of mobile health (mHealth) app has further facilitated the transfer of cognitive training from traditional clinical or laboratory settings to everyday life. Among these, cognitive training has shown potential in mitigating cognitive decline by engaging in specific exercises targeting various cognitive domains, such as memory, attention, processing speed, and executive function, to stimulate and enhance the connectivity and efficiency of brain neural networks [3]. A systematic review by Gates et al [4] found that cognitive training resulted in significant improvements in memory and attention in older adults with mild cognitive impairment, with long-term benefits persisting beyond the intervention period. Lampit et al [5] demonstrated that cognitive training in cognitively healthy older adults, particularly computerized interventions, resulted in moderate improvements in global cognition and specific domains such as processing speed and memory. Notably, a systematic search of the scientific literature was conducted in the Web of Science using a topic search with the terms “cognitive training” and “cognitive impairment.” The results show a steady increase in publications over the past 2 decades, as illustrated in Figure 1. Annual output rose from 58 papers in 2000 to over 1,000 per year after 2020, peaking at 1,091 in 2021. Despite slight declines in 2022 and 2023, the cumulative total reached 9,978 by 2023, reflecting sustained and growing academic interest in cognitive training for cognitive impairment.

In recent years, digital interventions have attracted growing attention as promising strategies to support cognitive health among older adults with cognitive impairment. A range of modalities, including mHealth apps, digital art therapy, and digital storytelling, have been explored with the aim of enhancing cognitive engagement and promoting rehabilitation [6-9]. Systematic and narrative reviews underscore both the potential benefits of these approaches and notable challenges related to accessibility, user engagement, and clinical validation [6,10]. Among these interventions, mHealth technologies have demonstrated particular promise by improving access to care, enabling real-time monitoring, and facilitating personalized health interventions [11,12]. Cognitive training apps delivered through mobile platforms offer greater accessibility, convenience, and cost-effectiveness compared to other cognitive training methods such as paper-and-pencil tasks, computer-based programs, or virtual reality–based interventions [13-15]. A growing number of cognitive training apps are now available, providing diverse exercises aimed at enhancing memory, attention, and executive function. Studies have shown that mHealth-based cognitive training apps can maintain or improve cognitive performance, especially when incorporating features such as adaptive difficulty levels, gamification, and feedback mechanisms to promote sustained engagement [16,17]. However, despite these advances, concerns remain regarding the methodological rigor, scientific validity, and evidence base underpinning many commercially available cognitive training apps. Recent reviews have highlighted substantial variability in app quality and the limited methodological rigor and standardization in existing evaluations of cognitive training apps [18,19]. Furthermore, it remains unclear to what extent current cognitive training apps have integrated recent innovations, such as individualized digital therapies and user-centered design principles. Therefore, a systematic and comprehensive quality evaluation of cognitive training apps is critically needed to inform future development and ensure these digital interventions truly meet the complex needs of specific population.

In the field of cognitive impairment, quality assessments of caregiver-related apps have been conducted [20]. Meanwhile, recent reviews have evaluated the content and quality of cognitive training apps available to the general population [18,19]; however, these evaluations often did not specifically target individuals with cognitive impairment. In addition, a recent scoping review by Silva et al [21] emphasized the substantial heterogeneity in intervention approaches among existing cognitive training apps and the lack of a standardized framework for app quality assessment. In order to optimize mobile apps, effectively meet user needs, and promote cognitive health, it is essential to establish a reliable metric for assessing app quality. Various tools have been developed to evaluate the quality of mHealth apps, such as the User Version of the Mobile App Rating Scale (uMARS) developed by Stoyanov et al [22], the Evaluation Tool for Mobile and Web-Based eHealth Interventions (ENLIGHT) developed by Baumel et al [23], and the System Usability Scale (SUS) developed by Brooke [24]. However, each of these instruments presents specific limitations. uMARS is primarily designed to capture feedback from general users, ENLIGHT focuses on behavioral change and lacks emphasis on broader usability and design elements, while SUS is limited to evaluating system usability alone. In contrast, the Mobile App Rating Scale (MARS), developed by Leanne’s research team, offers a simple, objective, and reliable tool for evaluating the overall quality of mobile apps. It assesses five core dimensions: engagement, functionality, aesthetics, information quality, and subjective quality, with clearly defined descriptors for each rating anchor to ensure consistency and accuracy [25]. MARS has been widely applied in assessing various health-related apps, including targeting genitourinary tumors, inflammatory bowel disease, psoriasis, and heart failure, with demonstrated reliability and applicability in related research contexts [26-29].

Given these gaps in the literature, the aim of this study was to identify and evaluate publicly available cognitive training apps designed for older adults with cognitive impairment. Specifically, this study aims to (1) identify and summarize existing cognitive training apps and their core features and (2) assess the quality of these apps using the MARS. It was hypothesized that existing cognitive training apps would exhibit variable quality and might not fully meet the specific needs of older adults with cognitive impairment. The findings from this study are expected to inform researchers, developers, and clinicians, contributing to the development of more effective, user-centered digital interventions for cognitive health promotion in this vulnerable population.

## Methods

### Study Design

Although the search strategy differed slightly from traditional methods, the study adhered to PRISMA (Preferred Reporting Items for Systematic Reviews and Meta-Analyses standards) Checklist present in Checklist 1 [30], and was consistent with recent reviews of mobile apps that applied similar methods [26,31]. A neuropsychologist from a large medical center and a certified software engineer oversaw and reviewed the research process and findings.

### App Search Strategy

A comprehensive search of the Google Play Store and Apple App Store was conducted up to July 13, 2024, using a 2-step approach, developed in collaboration with a librarian. In step 1, both app stores were searched with 67 relevant key terms (eg, “cognitive impairment,” “cognitive decline,” “brain,” “cognitive functions,” “cognitive therapy,” “cognitive training,” etc.). In step 2, string keywords were applied, combining multiple forms of cognitive impairment (eg, “cognitive dysfunction,” “cognitive declines,” “mental deterioration,” etc.) with terms such as “cognitive therapy,” “cognitive training,” “brain training,” etc. This approach accounted for differences in search algorithms: Apple’s optimized for single keywords and Google Play’s for string keywords. The details were presented in Multimedia Appendix 1. Relevant apps were identified based on the following inclusion criteria: (1) English language, (2) relevance to the subject matter, (3) free to download, (4) available for individual use, and (5) normal functionality.

### App Content Analysis

The primary content of the 24 representative apps was assessed by the two reviewers (LW and JP). These apps were required to focus primarily on cognitive training, targeting the enhancement and improvement of memory, attention, language, comprehension, and other cognitive functions.

### App Quality Evaluation

The quality of the included apps was evaluated using the MARS, a widely used tool developed by Stoyanov et al [25] in 2015 for evaluating mHealth apps. MARS has demonstrated high internal consistency (Cronbach α coefficient=0.90) and interrater reliability [25]. The scale consists of 23 items covering objective quality dimensions, including engagement, functionality, aesthetics and information, and subjective dimension. Specifically, engagement assesses whether the app is fun, interesting, customizable, interactive, and targeted; functionality assesses whether the app is easy to learn, easy to use, and logical; aesthetics assesses the graphic design, overall visual appeal, color scheme, and stylistic consistency of the app; information assesses whether the app contains high-quality information from credible sources; and the subjective quality dimension reflects user satisfaction, application adoption, and continuity of use.

Each item was scored on a 1-5 scale, with a “not applicable” option available. Following the MARS guidelines, the scoring procedure involved: (1) average score for each dimension for a single app is calculated by summing the scores of all items under that dimension and dividing by the total number of items in the dimension; (2) average score of each dimension across all apps was calculated by summing the dimension’s average scores for all apps and dividing by the number of apps; (3) overall quality score for a single app is determined by averaging the average scores of the four objective dimensions (engagement, functionality, aesthetics, and information), excluding the subjective quality dimension to maintain an unbiased evaluation; and (4) average overall quality score across all apps is calculated by summing the overall quality scores of all apps and dividing by the total number of apps. An average MARS score of ≥3 points (out of 5) is considered to be of “acceptable” quality [25].

### Data Collection

Two independent reviewers (LW and JP), both with medical backgrounds and specializing in cognitive impairment in older adults, conducted the app evaluations. Prior to the assessment, both reviewers received formal training in the use of the MARS to ensure consistency and accuracy. Each app was used for at least 10 minutes during evaluation to allow sufficient interaction with its functionalities. Apps were randomly assigned and independently evaluated without communication between reviewers to minimize potential bias. Discrepancies greater than one point on any subscale were resolved through discussion; if consensus could not be reached, a third reviewer (CD) was consulted to adjudicate the final score. Interrater reliability between the two reviewers was assessed using the intraclass correlation coefficient (ICC), and the agreement was good (ICC 0.965 [95% CI 0.92‐0.985]).

### Data Analysis

The two reviewers (LW and JP) reconfirmed the dates and entered the scores into a cloud-based Microsoft Excel spreadsheet, followed by the computation of descriptive statistics for each rating. The data analysis procedure consisted of the following steps: (1) calculating the mean score for each quality dimension for each app and calculating its variance, (2) adding the mean scores for the engagement, functionality, aesthetics, and information dimensions and dividing them by the average of the number of dimensions as the MARS final score and calculating its variance, (3) calculating the overall mean score for each dimension for all apps and calculating its variance, and (4) calculating the overall mean score by summing and dividing by the total number of apps to calculate the overall average score of all apps and calculate its variance. In addition, the apps were categorized into three groups based on the overall mean score: less than 3 (Group 1), 3-4 (Group 2), and greater than 4 (Group 3). The Kruskal-Wallis test was used to compare median scores across the three groups for each of the four MARS dimensions: engagement, functionality, aesthetics, and information, identifying significant variations among the subgroups. The Spearman correlation coefficient was used to assess the correlation between the star rating score and the overall mean score.

### Ethical Considerations

This study was exempt from institutional review board approval, as no risk to human participants was involved, and was registered at PROSPERO (International Prospective Register of Systematic Reviews, number CRD42024602240). This study involved a systematic search and quality assessment of publicly available mobile applications, conducted using the validated Mobile App Rating Scale (MARS). The study did not include the recruitment of human participants, collection of personal or health-related information, or any form of intervention involving individuals. All information was obtained from open-access app store listings and publicly available app descriptions. According to established ethical guidelines, including those outlined by institutional review boards (IRBs) in China and internationally (the Declaration of Helsinki, Council for International Organizations of Medical Sciences [CIOMS] guidelines), research that does not involve human participants or identifiable personal data is generally exempt from ethical review. Our study aligns with these criteria. Consistent with this, numerous high-impact, peer-reviewed studies involving app evaluations have also reported similar exemptions from ethical review, for example in references [32-36]. Based on this precedent and our institution’s policy, we confirm that no ethical approval was required for this study.

## Results

### App Selection

**Figure 2.**
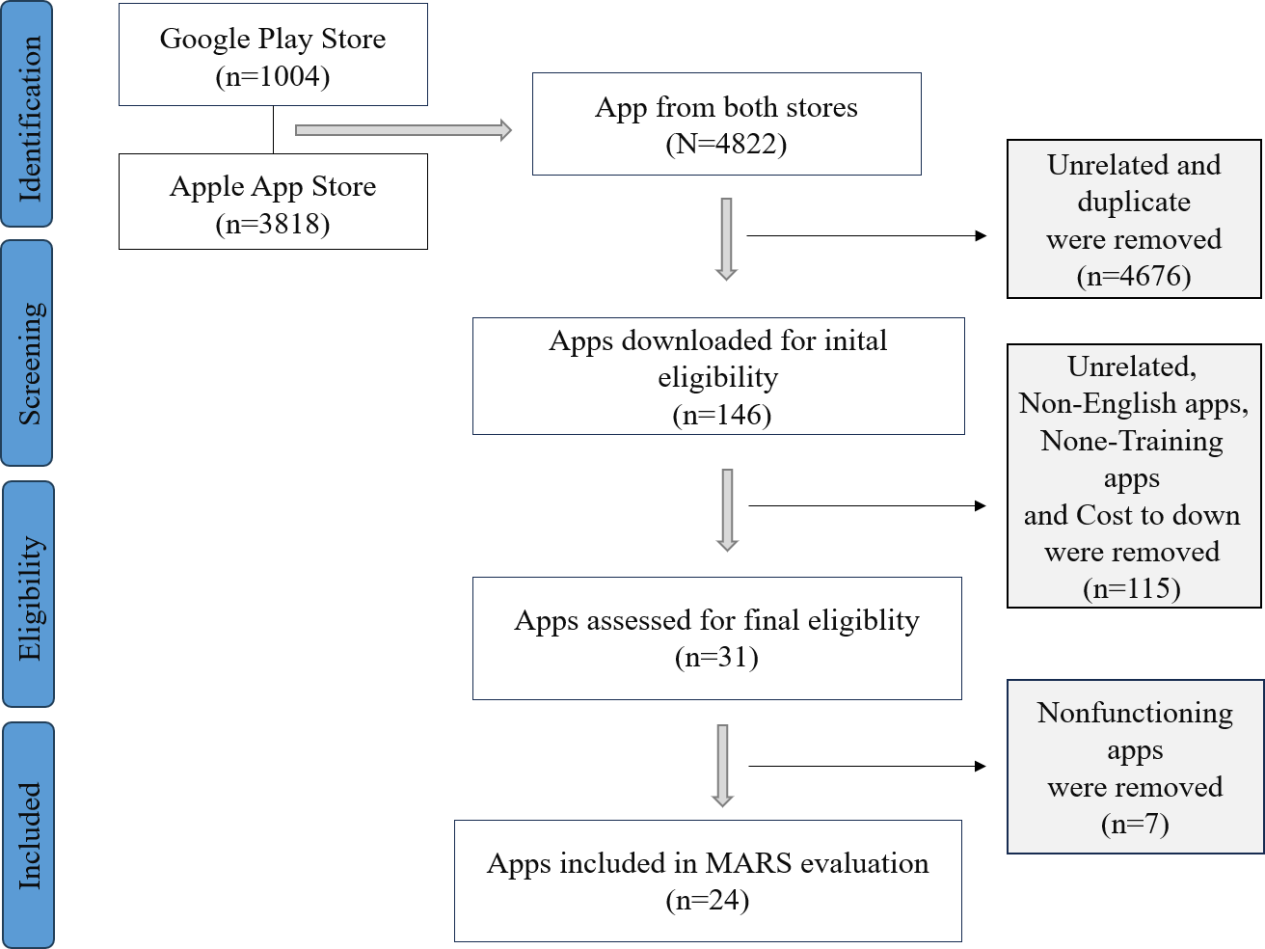
The process of apps selection. MARS: Mobile App Rating Scale.

A total of 4822 apps were retrieved from the Google Play Store and Apple App Store (1004 from Google Play Store and 3818 from Apple App Store). After preliminary screening, 146 apps were assessed for eligibility with duplicate and obviously irrelevant apps were removed. Following a detailed review, 31 apps were included, but 7 apps were found to be nonfunctioning, leaving 24 apps that met the inclusion criteria and were eventually included for evaluation as shown in Figure 2.

### Characteristics and Purposes of Included Apps

Of these 24 apps, 13 (54%) are available on both platforms, 5 (21%) are exclusive to the Google Play Store, and 6 (25%) are accessible on the Apple App Store. Table 1 outlines the content characteristics of each app. The primary focus of the included apps is cognitive training aimed at improving memory, attention, language comprehension, and other cognitive functions. In total, 5 apps (20.8%) allow users to select training modules tailored to their individual needs. Overall, 8 apps (33.3%) indicate the involvement of medical and related professionals in their development process, as stated in the app profiles or within the apps.

**Table 1.** Description of the apps included in the study.

App name	Platform	Developer	Age group (years)	Involvement of health care	Focus (targeted areas of the app)
Memory Trainer	iOS	Svyatoslav Ivashchenko	4 years or older	Unknown	Memory
Brave	iOS	University of Hong Kong	17 years or older	Unknown	MCI^a^ education and related physical training
Dementia Learning Game	iOS	Bom Soft	4 years or older	Unknown	Concentration, observation,and memory
Mindmate	iOS	Mindmate Ltd	12 years or older	Unknown	Problem-solving, attention, and lifestyle support
Cognitive Skill Training	iOS	Kazuaki Matayoshi	4 years or older	Unknown	Arithmetic, memory, and attention
Reminding	iOS	Sync International Pty Ltd	12 years or older	Unknown	Game-based cognitive training
Mindpal	Android	Elektron Labs Inc	All ages	Unknown	Language, memory, attention, and problem-solving
Elevate	Android	Elevate Labs	All ages	Yes	Attention, language, memory, processing speed, and math
Neurobics	Android	Peoresnada.com	All ages	Unknown	Mental, attention, calculation, and memory, analysis
Train Your Brain Memory Games	Android	Senior Games	All ages	Yes	Memory-focused cognitive stimulation
Abrain	Android	Abrain Labs	All ages	Unknown	Memory, attention, reaction, and math
Cognishape	iOS and Android	Cognishape	12 years or older	Yes	Attention, memory, creativity, and problem-solving
Q4 Active - Brain Health	iOS and Android	Genius Gyms LLC	4 years or older	Yes	Cognitive enhancement and decline prevention
Brain HQ	iOS and Android	Posit Science	4 years or older	Yes	Cognition, function, and brain plasticity
Alzlife	iOS and Android	Alzheimer’s Light	17 years or older	Unknown	Sensory stimulation and cognitive games
Ginkgo Memory & Brain Training	iOS and Android	Ginkgo Academy	4 years or older	Unknown	Cognition and memory retention
Recover Brain	iOS and Android	Imagiration LLC	4 years or older	Yes	Memory, language, comprehension and executive
Lumosity	iOS and Android	Lumos Labs, Inc.	4 years or older	Unknown	Attention, flexibility, and problem-solving
Constant Therapy: Brain Rehab	iOS and Android	Constant Therapy, Inc	12 years or older	Yes	Language, memory, attention, reading, math, and comprehension
Memorie	iOS and Android	Neeuro Pte Ltd	4 years or older	Unknown	Attention, memory, decision-marking, and spatial perception
Focus - Train Your Brain	iOS and Android	Tellmewow	4 years or older	Unknown	Memory, coordination, and attention
Impulse	iOS and Android	Gmrd Apps Limited	4 years or older	Unknown	Memory, attention, and concentration
Peak	iOS and Android	Synaptic Labs	4 years or older	Yes	Brain assessment, memory, attention, and language
Neuronation	iOS and Android	Synaptikon Gmbh	4 years or older	Unknown	Memory and concentration

a MCI: mild cognitive impairment

### MARS Evaluation of Included Apps

The 24 included apps were evaluated by two reviewers, with substantial interrater agreement (Quadratic Weighted κ=0.88, 95% CI 0.80‐0.95), indicating a high level of agreement between the 2 reviewers. Based on the MARS evaluation, apps quality was assessed across four dimensions: engagement, functionality, aesthetics, and information, shown in Table 2. The overall mean score was 3.57 (SD 0.43), indicating that the apps were generally acceptable. Furthermore, 2 apps, Brain HQ and Peak, received scores above 4 and were rated as good, while 3 apps, Memory Trainer, Cognitive Skill Training, and Ginkgo Memory & Brain Training, scored below 3 and were considered insufficient. Among the 4 dimensions, functionality had the highest mean score, while engagement scored the lowest. In addition, the Spearman correlation showed no significant relationship between the overall mean score and star rating (*R*=−0.331, *P*=.18).

**Table 2.** The mean (SD) scores of each Mobile App Rating Scale dimension for included apps.

App name	Engagement	Functionality	Aesthetics	Information	Overall score	Star rating score
Memory Trainer	1.4 (0.49)	5 (0)	2 (0)	1.75 (0.43)	2.54 (1.44)	^a^ ^—^
Brave	3 (1.1)	4.75 (0.43)	4 (0.82)	4.2 (0.4)	3.99 (0.63)	—
Dementia Learning Game	3.2 (1.17)	4.25 (0.43)	3.67 (0.47)	3.5 (0.5)	3.65 (0.38)	—
Mindmate	3.2 (0.98)	4.5 (0.5)	3.67 (0.47)	3.8 (0.4)	3.79 (0.47)	5
Cognitive Skill Training	2.6 (1.02)	3.5 (0.5)	3 (0.82)	2.75 (0.43)	2.96 (0.34)	4.5
Reminding	2.6 (0.49)	4 (0)	3.33 (0.47)	3.5 (0.5)	3.36 (0.5)	—
Mindpal	3.6 (0.49)	4.5 (0.5)	3.67 (0.47)	3.6 (0.49)	3.84 (0.38)	4.5
Elevate	3.2 (0.4)	4.5 (0.5)	3.33 (0.47)	3.33 (0.47)	3.59 (0.53)	4.6
Neurobics	3.6 (0.49)	4.5 (0.5)	3.33 (0.47)	3.6 (0.49)	3.76 (0.44)	4
Train Your Brain Memory Games	3 (0.89)	4.25 (0.83)	4 (0)	3.25 (0.43)	3.63 (0.52)	4.5
Abrain	3.4 (0.49)	4.25 (0.43)	3.33 (0.47)	3.4 (0.49)	3.60 (0.38)	4.5
Cognishape	2.4 (0.49)	4.25 (0.43)	3 (0.82)	3.25 (0.43)	3.23 (0.67)	4.1
Q4 Active-Brain Health	2.6 (0.49)	4 (0.71)	3.33 (0.47)	3 (0)	3.23 (0.51)	—
Brain HQ	4 (0.63)	4.5 (0.5)	4 (0)	4 (0.58)	4.13 (0.22)	3.7
Alzlife	3.8 (1.17)	4 (0)	3.67 (0.47)	3.67 (0.47)	3.78 (0.14)	4.2
Ginkgo Memory& Brain Training	2 (0)	2.25 (0.83)	2.67 (0.47)	2.6 (0.49)	2.38 (0.27)	4.6
Recover Brain	3.6 (0.49)	4.5 (0.5)	4 (0)	3.8 (0.4)	3.98 (0.33)	4.2
Lumosity	3.2 (0.98)	4.25 (0.43)	4 (0)	3.43 (0.49)	3.72 (0.42)	4.2
Constant Therapy: Brain Rehab	3.4 (0.49)	4.5 (0.5)	4 (0)	3.6 (0.49)	3.88 (0.42)	4.15
Memorie	3 (1.26)	4.25 (0.43)	3.33 (0.47)	3.6 (0.49)	3.55 (0.46)	—
Focus - Train Your Brain	3.6 (0.49)	4.5 (0.5)	3.67 (0.47)	3.4 (0.49)	3.79 (0.42)	4.8
Impulse	3.4 (1.36)	4.25 (0.43)	4 (0)	3.4 (0.8)	3.76 (0.37)	4.5
Peak	4 (0.89)	4.75 (0.43)	4 (0)	3.6 (0.49)	4.09 (0.42)	4.3
Neuronation	2.6 (0.49)	4.5 (0.5)	3.33 (0.47)	3.2 (0.4)	3.41 (0.69)	4.55
Total (SD)	3.1 (0.61)	4.27 (0.51)	3.51 (0.49)	3.38 (0.48)	3.57 (0.43)	—

a Not applicable.

**Figure 3.**
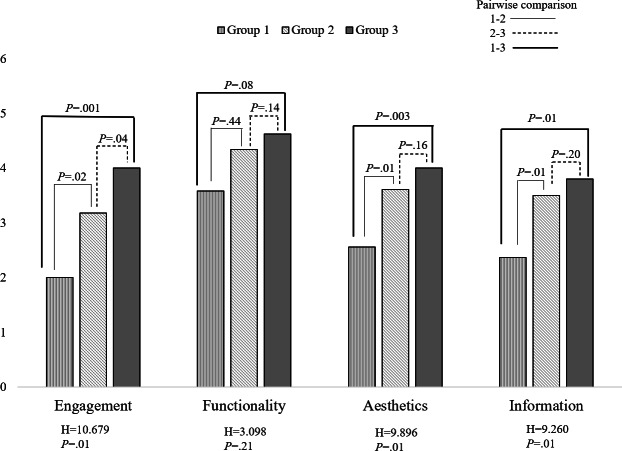
Group differences in MARS dimension scores. MARS: Mobile App Rating Scale.

The included apps were categorized into three groups based on their overall mean scores: less than 3 (Group 1), between 3 and 4 (Group 2), and greater than 4 (Group 3). Detailed descriptions of these groupings are provided in Multimedia Appendix 2. The H value refers to the Kruskal–Wallis test statistic that indicates the degree of difference in rank sums among groups; higher values suggest greater group differences. Figure 3 shows the differences in MARS dimension scores between groups. No significant difference was observed in the functionality dimension (*P*=.21); however, differences in all other dimensions were statistically significant, with the engagement dimension showing the largest difference (*P*=.01). Pairwise comparisons revealed the following: (1) engagement: significant differences were observed between all groups, with score differences of 1.18 (Group 1 vs Group 2, *P*=.02), 0.82 (Group 2 vs Group 3, *P*=.04), and 2.00 (Group 1 vs Group 3, *P*=.001); (2) aesthetics: significant differences were found between Group 1 versus Group 2 (score difference=1.05, *P*=.01) and Group 1 versus Group 3 (score difference=1.44, *P*=.003), but not between Group 2 versus Group 3; (3) information: significant differences were noted between Group 1 versus Group 2 (score difference=1.13, *P*=.01) and Group 1 versus Group 3 (score difference=1.43, *P*=.01), with no difference between Group 2 versus Group 3.

Bivariate Spearman correlation was calculated to test the relationship between the overall mean score and the star rating score. The results show that the overall mean score and the star rating score are not statistically significantly correlated (*R*=−0.331; *P*=.18).

## Discussion

### Principal Findings

Compared to previous studies that reviewed cognitive training apps primarily for the general population [18,19], this study is the first to systematically evaluate apps specifically designed for individuals with cognitive impairment, a population with distinct cognitive and usability needs. By applying the MARS to assess 24 apps across multiple quality dimensions, this study provides a comprehensive, standardized evaluation of app strengths and limitations. Overall, the apps demonstrated an acceptable level of quality. The functionality dimension received the highest scores, indicating strong usability and practicality, whereas the engagement dimension scored the lowest, reflecting limited user interactivity. Significant variability was observed across apps in engagement, aesthetics, and information quality. Furthermore, the findings highlight critical deficiencies in user-centered design and the limited involvement of health care professionals in app development. These insights identify key areas for improvement and offer important guidance for developers and health care providers seeking to optimize cognitive health interventions for individuals with cognitive impairment.

The quality of the 24 cognitive training apps was assessed using the MARS, with an average score of 3.57 (SD 0.43). Most apps (79.2%) were rated as acceptable, while only a small proportion (8.3%) received a good rating. Among the 4 MARS dimensions, functionality scored the highest, reflecting intuitive navigation, reliable performance, and device integration. In contrast, engagement received the lowest scores, followed by information quality, indicating a lack of features that sustain user interest and provide credible, evidence-based content. This quality pattern has also been reported in evaluations of other mHealth apps. Carrouel et al [37] evaluated mental health apps and found that although most demonstrated sound technical performance, many lacked meaningful user engagement and evidence-based content. Similarly, Martinon et al [38] reported that nutrition-related apps were generally usable but underperformed in terms of personalization and information quality. These similarities suggest a broader trend in mHealth app development, where technical functionality often exceeds performance in user-centered and content-related dimensions. As earlier research has pointed out, low engagement scores in health-related apps are often linked to a lack of personalized and adaptive content, which is particularly important for diverse populations such as older adults with cognitive impairments [39]. Furthermore, low engagement is often linked to poor user retention. In one study on diabetes management apps, low engagement scores were associated with higher dropout rates [40]. Therefore, incorporating personalized features and adaptive content, such as gamification, user incentives, and social interaction tools, is essential to enhancing engagement and ensuring sustained use of cognitive training apps.

This study further revealed that group comparisons of the 4 MARS dimensions showed no significant differences in functionality; however, significant differences were observed in engagement, aesthetics, and information across app groups. The consistent functionality suggests that most cognitive training apps meet baseline standards for technical performance, reliability, and ease of use. In contrast, the observed differences in engagement, aesthetics, and information highlight key areas where apps diverge in their ability to meet user needs. Variations in engagement likely reflect differing levels of investment in user-focused features [41]. Differences in aesthetics and information may indicate disparities in design quality and content accuracy, with higher-scoring apps often incorporating professional input and evidence-based content [42,43]. Addressing these gaps by enhancing engagement features, improving design, and involving professionals during development could enhance app effectiveness and user satisfaction. Notably, limited involvement of health care professionals during app development for older adults with cognitive impairment, as observed in this study, is consistent with previous findings [27,44]. Only 8 of the included apps (33%) explicitly mentioned health care professional involvement. This reflects a broader challenge in the mHealth field [45], where insufficient engagement from both health care professionals and patients often results in apps that fail to provide reliable, evidence-based information or meet user needs effectively. Evidence indicates that the quality and trustworthiness of mHealth apps are strongly linked to the involvement of health care professionals, who ensure clinical accuracy and adherence to evidence-based practices [46]. Similarly, excluding patients from the development process frequently leads to features misaligned with real-world needs, reducing engagement and limiting the apps’ effectiveness in supporting health management [47].

The findings of this study have important implications for both app developers and health care providers. Several key deficiencies were identified across many of the reviewed apps, including user engagement, information quality, aesthetic design, and the limited involvement of health care professionals, which underscores the need for closer collaboration with health care professionals to ensure content accuracy, adherence to medical guidelines, and alignment with real-world health care needs [46,48]. Similarly, incorporating patient feedback during the development process can help tailor cognitive training apps to better meet the cognitive, physical, and sensory needs of older adults with cognitive impairment, thereby improving accessibility and engagement. Furthermore, the results of this study also underscore the importance of user interface design, balancing aesthetic appeal with intuitive functionality can significantly enhance usability, particularly in cognitively impaired populations. On the technical side, integrating technical features such as adaptive difficulty levels and real-time feedback could enhance personalization and maintain user motivation over time. Overall, these insights underscore the value of a cross-disciplinary approach in which developers, clinicians, patients, designers, and technical teams collaborate to produce mHealth solutions that are user-centered, clinically credible, and sustainable in real-world use.

This study identified key limitations in existing cognitive training apps for individuals with cognitive impairments, particularly in visual presentation, such as dense text blocks and a lack of instructional images and videos, which are particularly challenging for this population. As cognitive impairments often lead to age-related visual degradation, including decreased visual acuity, reduced field of vision, and diminished color recognition, these issues exacerbate difficulties in using digital technologies, especially for cognitive training [49]. Previous studies indicated that cognitive impairments also introduce unique visual challenges, such as slower visual processing speed and difficulties in spatial perception, which hinder the effective use of apps [50,51]. To improve the usability and effectiveness of cognitive training apps for older adults with cognitive impairment, it is essential to incorporate user-centered design strategies that accommodate both cognitive and sensory limitations. Interface design should emphasize simplicity and clarity through the use of high-contrast visuals, large fonts and icons, linear navigation structures, and minimal interaction steps to reduce operational complexity and cognitive load. Readability can be further enhanced by optimizing text presentation with adjustable font sizes, low content density, and voice-assisted reading or playback features to support information processing and minimize visual fatigue. Furthermore, incorporating multimodal support, such as auditory prompts, visual cues, haptic feedback, and the inclusion of icons, images, and instructional videos can enhance task comprehension and user engagement. In particular, the integration of auditory and visual stimuli has been shown to improve performance and reduce cognitive burden [52]. To further support visual processing and maintain attention, optimizing visual presentation (such as enhancing contrast and minimizing screen glare) is also important, as higher contrast improves readability while glare reduction contributes to visual comfort and uninterrupted use [53,54]. In addition, personalized cognitive training modules that adapt to users’ cognitive profiles, including their impairment levels, response speed, learning preferences, and progress over time, may help maintain motivation and optimize training outcomes. Finally, enhancing feedback mechanisms is also critical. Real-time audio cues, digital guidance, and multisensory reinforcement, including auditory and tactile feedback, can promote sustained attention and support memory retention [55]. These strategies will facilitate the improvement of usability and effectiveness in cognitive training apps for older adults with cognitive impairments, enhancing engagement and supporting better cognitive performance.

### Limitations

Several limitations should be considered in this study. First, data were sourced primarily from major app stores (Google Play Store and Apple App Store), which may have excluded apps available on regional or specialized platforms, limiting the comprehensiveness of the findings. Second, although the MARS provided a standardized evaluation framework, inherent subjectivity in the scoring process may have affected consistency, particularly in the evaluation of visual design. Third, the study focused exclusively on apps identified using specific cognitive training-related keywords, resulting in a limited sample size and potentially reducing the generalizability of the findings. Fourth, the dynamic nature of app stores posed challenges to the study’s timeliness, as some apps may have been removed or updated after data collection. Finally, while MARS is a robust tool, it does not comprehensively address data privacy concerns or sustained app usage outcomes. Future research should address these limitations by expanding data sources, incorporating user experience and longitudinal data, and exploring additional domains of app quality.

### Conclusions

This study evaluated the quality of cognitive training mobile apps for older adults with cognitive impairment using the MARS. The findings highlight the promise of these apps as accessible tools for cognitive health management while revealing notable deficiencies in quality, usability, and evidence-based functionality. As mHealth technologies evolve, rigorous and standardized evaluation frameworks and longitudinal studies are needed to validate their efficacy and ensure safety in real-world settings. Such efforts are critical to fully realize the potential of these technologies in supporting cognitive health in aging populations.

## Supplementary material

10.2196/69637 Multimedia Appendix 1 Search Strategy Process.

10.2196/69637 Multimedia Appendix 2 Grouping.

10.2196/69637 Checklist 1 PRISMA (Preferred Reporting Items for Systematic Reviews and Meta-Analyses) checklist.

## Abbreviations

ENLIGHT: Evaluation Tool for Mobile and Web-Based eHealth Interventions
ICC: intraclass correlation coefficient
MARS: Mobile App Rating Scale
mHealth: mobile health
PRISMA: Preferred Reporting Items for Systematic Reviews and Meta-Analyses
PROSPERO: International Prospective Register of Systematic Reviews
SUS: System Usability Scale
uMARS: User Version of the Mobile App Rating Scale
