# Supplementary material for: Cognitive Training Mobile Apps for Older Adults With Cognitive Impairment: App Store Search and Quality Evaluation
Source: JMIR Mhealth Uhealth. 2025 Jul 4;13:e69637. doi: 10.2196/69637 (PMC12252145; doi:10.2196/69637)
Supplement: Multimedia Appendix 1 [file mhealth-v13-e69637-s001.docx]

**Applying Search Strategies**

**Search Objective:**

The objective of this search was to identify apps related to cognitive training for older adults with cognitive impairment, and we focused on relevant apps in two major app stores, the Apple App store and Google play store.

**Search the App Store:**

1. Apple App store

2. Google play store

**Retrieval timeframe:** time until 2024.7.13.

**Search Strategies:**

1. Apple app store:

- Platform of use: iPhone, iPad devices or via the App Store website (https://www.apple.com/app-store/).

2. Google play:

- Platform of use: Android devices or through the Google Play website (https://play.google.com/).

**Search objective:** To find applications related to cognitive training for older adults with cognitive impairment.

**Keyword selection:** search the app store for relevant keywords or phrases, e.g., brain, cognitive impairment, etc., as shown in the attached [**indexed vocabulary**](#A).

**inclusion criteria:**

- English language

- relevance to the subject matter

- free to download

- available for individual use

- normal functionality

**search step:**

1. Search with related keywords

2. String keywords were applied, combining multiple forms of cognitive impairment (e.g., ‘cognitive dysfunction’, ‘cognitive declines’, ‘mental deterioration’, etc.) with terms such as ‘cognitive therapy’, ‘cognitive training’, ‘brain training’, etc.

3. Extract information about the app in question, including app name, developer, star ratings, specifics, etc.

**Search Results:**

- The Apple App Store returned 1004 relevant apps and Google Play returned 3818 relevant apps. After initial screening, 146 apps passed the eligibility assessment after removing duplicates and obviously irrelevant apps. Following a detailed review, 31 apps were included, but 7 were found to be inoperable, resulting in 24 apps meeting the criteria to be included.

**indexed vocabulary**

| **Brain** |
| --- |
| MeSH Entry Terms: |
| Encephalon |
| **Cognitive Impairment** |
| MeSH Entry Terms |
| Cognitive Dysfunctions |
| Dysfunction, Cognitive |
| Dysfunctions, Cognitive |
| Cognitive Disorder |
| Cognitive Disorders |
| Disorder, Cognitive |
| Disorders, Cognitive |
| Cognitive Impairments |
| Impairment, Cognitive |
| Impairments, Cognitive |
| Cognitive Decline |
| Cognitive Declines |
| Decline, Cognitive |
| Declines, Cognitive |
| Mental Deterioration |
| Deterioration, Mental |
| Deteriorations, Mental |
| Mental Deteriorations |
| **Cognition** |
| MeSH Entry Terms: |
| Cognitions |
| Cognitive Function |
| Cognitive Functions |
| Function, Cognitive |
| Functions, Cognitive |
| **Cognitive Behavioral Therapy** |
| MeSH Entry Terms: |
| Behavioral Therapies, Cognitive |
| Behavioral Therapy, Cognitive |
| Cognitive Behavioral Therapies |
| Therapies, Cognitive Behavioral |
| Therapy, Cognitive Behavioral |
| Cognition Therapy |
| Cognition Therapies |
| Therapies, Cognition |
| Therapy, Cognitive Behavior |
| Behavior Therapies, Cognitive |
| Cognitive Behavior Therapies |
| Therapies, Cognitive Behavior |
| Therapy, Cognition |
| Behavior Therapy, Cognitive |
| Cognitive Behavior Therapy |
| Cognitive Psychotherapy |
| Cognitive Psychotherapies |
| Psychotherapies, Cognitive |
| Psychotherapy, Cognitive |
| Therapy, Cognitive |
| Cognitive Therapies |
| Therapies, Cognitive |
| Cognitive Behaviour Therapy |
| Behaviour Therapies, Cognitive |
| Behaviour Therapy, Cognitive |
| Cognitive Behaviour Therapies |
| Therapies, Cognitive Behaviour |
| Therapy, Cognitive Behaviour |
| Cognitive Therapy |
| **cognitive rehabilitation** |
| MeSH Entry Terms: |
| Cognitive Training |
| Training, Cognitive |
| Rehabilitation, Cognitive |
| Brain Training |
| Training, Brain |
| Memory Training |
| Training, Memory |
| Cognitive traning |
| **Cognitive intervention** |
